# Supplementary material for: Unidirectional ion transport in nanoporous carbon membranes with a hierarchical pore architecture
Source: Nat Commun. 2021 Jul 30;12:4650. doi: 10.1038/s41467-021-24947-3 (PMC8324873; doi:10.1038/s41467-021-24947-3)
Supplement: Supplementary file 1 — Supporting information [file 41467_2021_24947_MOESM1_ESM.pdf]

Supporting information for

Unidirectional ion transport in nanoporous carbon membranes with a hierarchical pore architecture

Lu Chen et al.

	Purge	Anneal	Grow	Cool
Temperature (℃)	1000	1000	1000	0
Time (min)	120	30	60	400
Argon (sccm)	50	50	50	50
Hydrogen (sccm)	-	-	+	-
Pressure (atm)	1	1	1	1

Supplementary Table 1. The detailed condition for the CMHPA fabrication, in which process the valve 3 is closed, where sccm means “standard cubic centimeters per minute”.

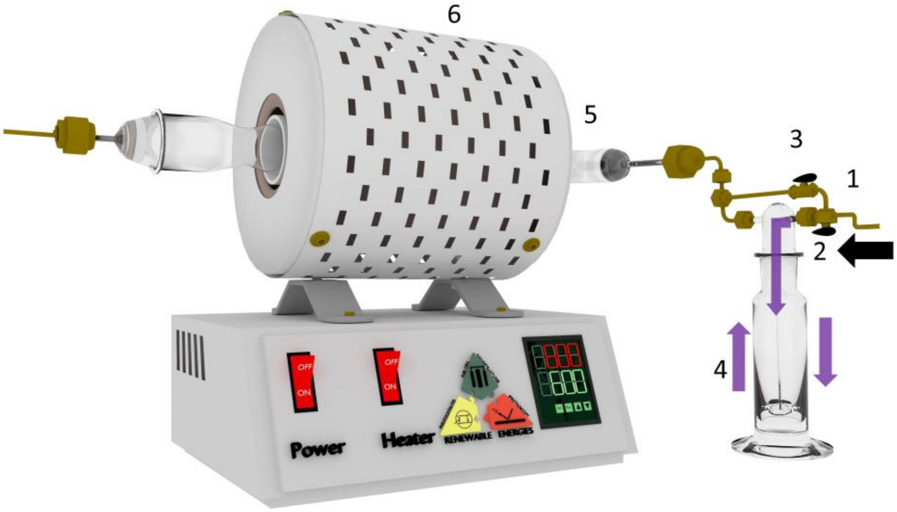

Supplementary Figure 1. Schematic of the CVD device. 1) Gas mixing: Ar, or Ar + H_2, Ar + H_2 + CH_4; 2) Changeover valve; 3) Changeover valve; 4) Liquid precursor; 5) Quartz tube; 6) APCVD.

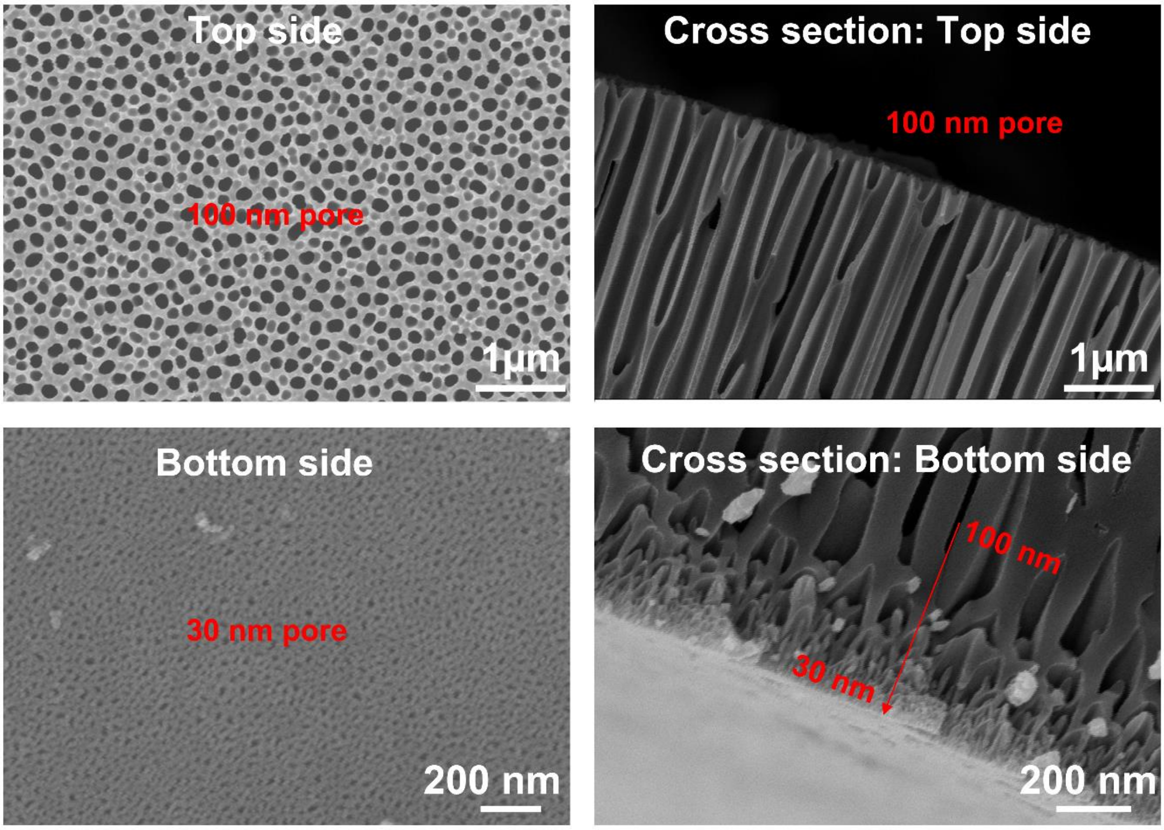

Supplementary Figure 2. Morphology characterizations of AAO substrate. The asymmetric anodic aluminum oxide (AAO) membrane with a pore diameter of 100 nm (large side) and 30 nm (narrow side) is used as a substrate.

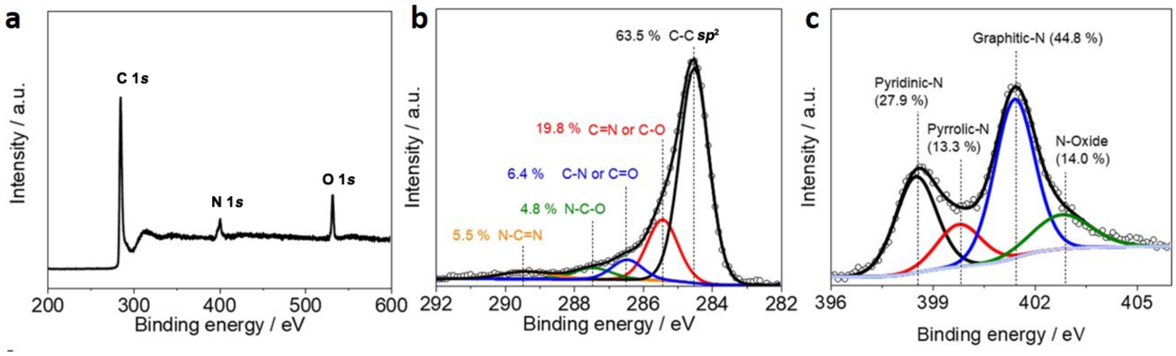

Supplementary Figure 3. Element characterizations of CMHPA. **a**, XPS survey of CMHPA; **b**, High-resolution C 1*s* spectra of CMHPA, indicating typical C–C, C–N, and C–O bonds; **c**, High-resolution N 1*s* spectra of CMHPA, indicating the formation of pyridinic-N, pyrrolic-N, graphitic-N, and oxidized-N species.

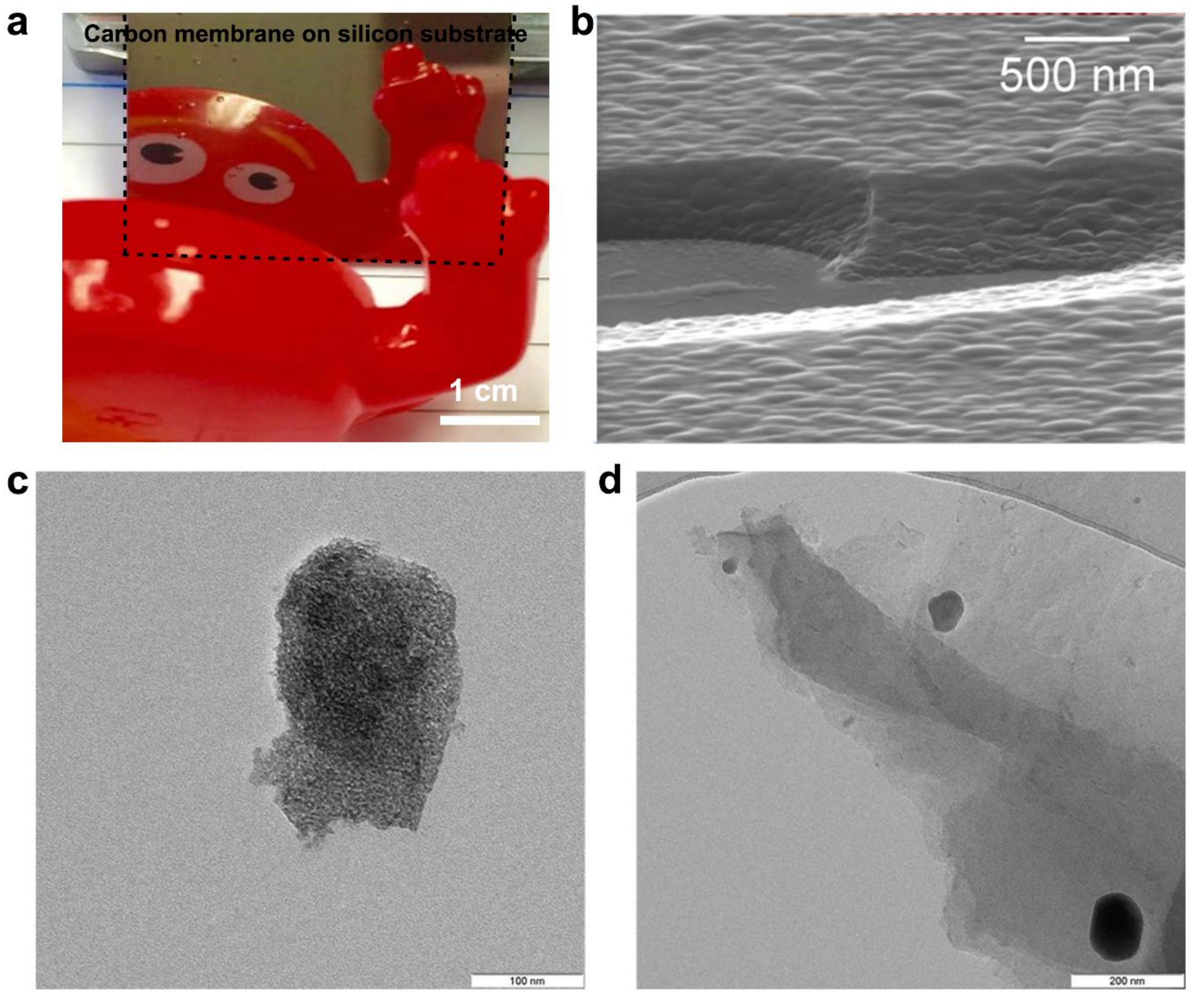

Supplementary Figure 4. Morphology characterizations of CMHPA. a, Metallic black carbon membrane on silicon substrate. **b,** SEM image of black carbon membrane on silicon substrate. **c, d,** TEM images of metallic black carbon, indicating the layered and porous structure.

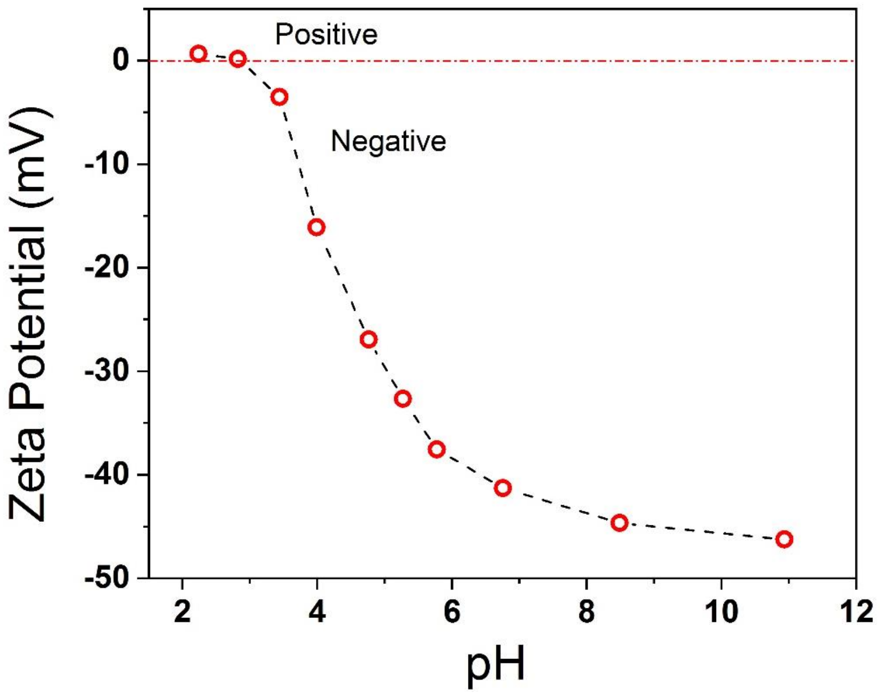

Supplementary Figure 5. Zeta-potential of CMHPA. The pK_a is estimated between 2.8 and 3.5.

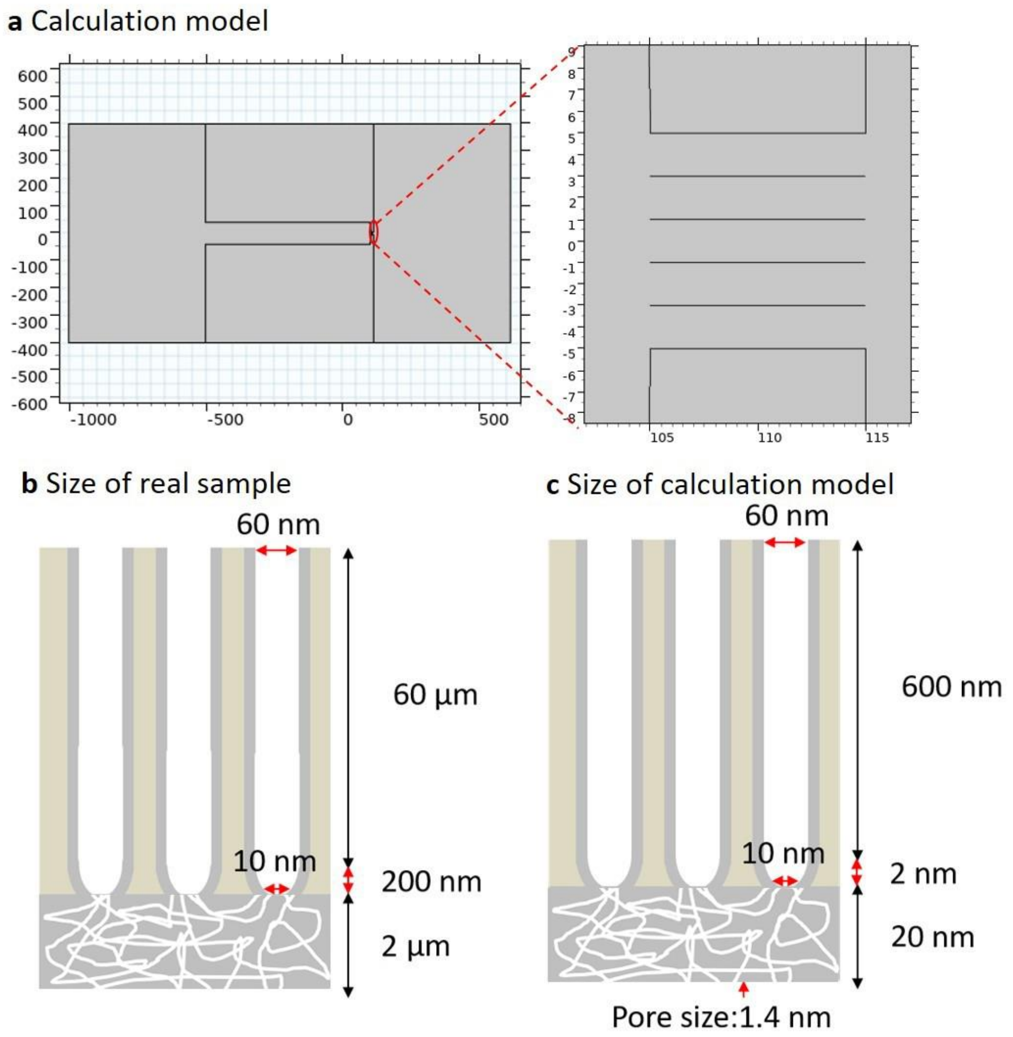

Supplementary Figure 6. Calculation parameters. a, Calculation model of the CMHPA. **b**, Size of the real sample. **c**, Size of the simplified calculation model. For simplicity, we use a 600-nm-length cylindrical nanochannel and a 20-nm-thick membrane to simulate the real case.

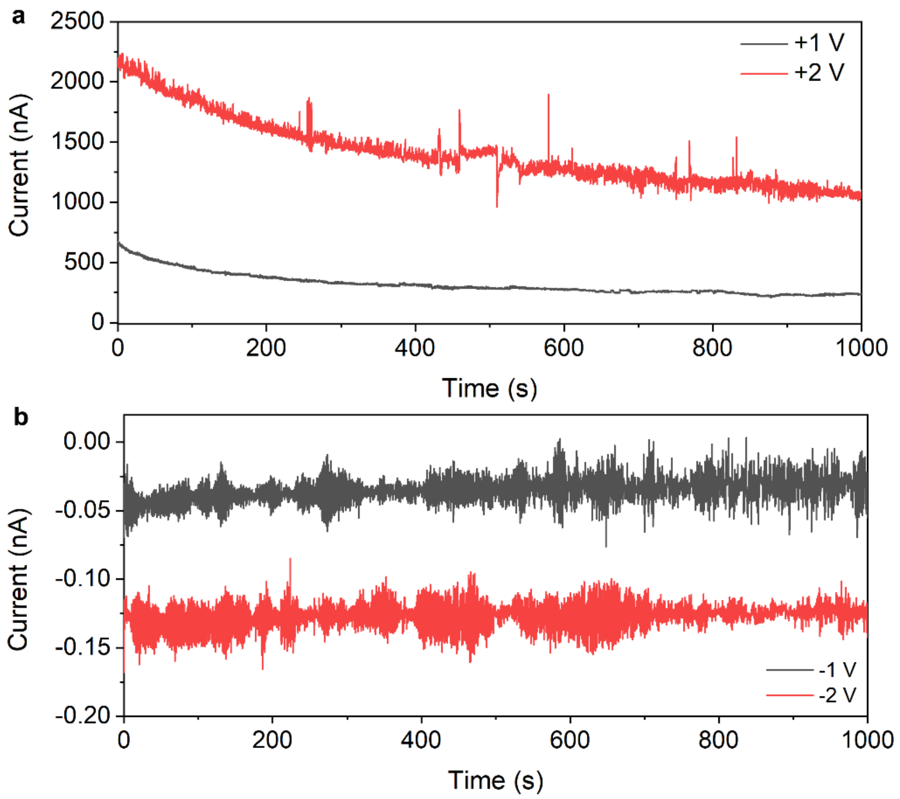

Supplementary Figure 7. Stability characterization. a, The constant current under +1 V and +2 V. **b**, The constant current under -1 V and -2 V. The carbon membrane after high-temperature deposition shows robust ion transport stability. The ionic currents both under positive and negative voltage are stable in 1000 s, despite of the attenuation in the first 300 s under positive voltage because of electrode reaction. Excellent stability provides a good vision for its further application.

Supplementary methods

1. **Experimental section:**

General: Unless otherwise noted, all of the commercial reagents were used as received. Target 60-μm-thick AAO membranes were purchased from Heifei Puyuan Nano, China. LED light were used for light irradiation. In this work, unless otherwise noted, all the light illumination was provided by white LED light. *I-V* curves and constant voltage ionic current were adjusted to zero current at zero voltage to remove small offsets experienced between runs. All measurements were carried out at room temperature. The main transmembrane potential used in this work was stepped at 1 s/step, with its period of 21 s. For example, the scanning voltage from -1V to +1V has a scanning step 0.1 V/step/s. For the scanning voltage from -10V to +10V, the voltage step should be 1 V/step/s.

Fabrication of CMHPA. For the fabrication of CMHPA, a standard atmospheric pressure CVD (APCVD) was employed using a commercial warm-wall reactor. Porous anodic aluminum oxide (AAO) was used as the growth substrate. The CMHPA synthesis procedure, shown in Figure 1 and Table S1, consisted of an annealing step for 30 min at 1000 ℃ with argon (Ar) 50 sccm, followed by a growth step for 60 min at 1 atm with an Ar and humidified with acetonitrile (ACN) (Alfa Aesar, 99%) bubbler. In a final step, the system was allowed to cool down passively and an Ar flow rate of 50 sccm, reaching less than 30 ℃ in ca. 400 min.

Ionic transport properties. The ions transport properties were studied by measuring the current-voltage curves and constant voltage ionic current through the carbon membrane, which was mounted between two chambers of a home-made H cell, which are full of electrolytes. The cell has a transparent glass window for light irradiation. Ag/AgCl electrodes were used to collect the current and voltage signals. Ionic current (photocurrent) was measured by a Keithley 6430 picoammeter (Keithley Instruments, Cleveland, OH). Light power intensity was measure by a portable light intensity meter.

Calculation. The trans-nanotube potential was systematically analyzed by a theoretical model based on Poisson and Nernst-Planck (PNP) equations with proper boundary conditions,

$$\nabla^2\Phi=-\frac{F}{\varepsilon}\sum z_i c_i$$

Supplementary Equation 1

$$j_i = D_i\nabla c_i + \frac{z_i F}{RT} D_i c_i \nabla\Phi$$

Supplementary Equation 2

$$\nabla \cdot j_i = 0$$

Supplementary Equation 3

Where Φ, c_i, D_i, j_i, z_i are, respectively, the electrical potential, ion concentration, diffusion constant, ionic flux, and charge of species i. ε is the dielectric constant of the electrolyte solution. The diffusion coefficients for cations and anions are 2.0×10^{-9} m^2 s^{-1} (we use KCl electrolyte for simplicity). The boundary condition for potential Φ on the nanotube wall is,

$$\vec{n}\cdot\nabla\Phi = -\frac{\sigma}{\varepsilon}$$

Supplementary Equation 4

where σ is the surface charge density. And the surface charge density is various along with our experiment condition. The ion flux has zero normal components at boundaries,

$$\vec{n}\cdot j = 0$$

Supplementary Equation 5

The geometric parameters are in Fig. S5. The stationary solver was generally used. But when it fails, the parametric solver was applied. For all the calculations, the accuracy is set to be less than 10^{-6}.

2. Characterizations

The X-ray photoelectron spectra were collected using monochromatic AlKα radiation (Kratos, Axis Ultra) and an Omicron DAR 400 X-ray source with an Al Kα line (15 kV) and an EA 125X hemispherical energy analyser (Omicron). The binding energies were referenced to the C1s line at 284.8 eV from adventitious carbon. The scanning electron microscope (SEM) JSM-7500F (JEOL) at an accelerating voltage of 3 kV was used to get the top view and cross-section of the membrane. The TEM is a double-corrected Jeol ARM200F, equipped with a cold field emission gun. The acceleration voltage was set to 200kV, and the emission was put to 10 μA. The Zeta Potential was measured with SurPASS 3, Anton Paar.

Pore structure properties of the samples were measured via nitrogen adsorption and desorption at 77 K using a volumetric technique on a Quantachrome Quadrasorb SI porosimeter. Prior to analysis, the carbons were degassed under vacuum at 200 ℃ for 12 h. Brunauer-EmmettTeller (BET) surface area was calculated in the relative pressure P/P0 < 0.2. Total pore volume (Vt) was determined from the amount of nitrogen adsorbed at P/P0=0.995. The pore size distributions of the carbons were obtained by quenched solid density functional theory (QSDFT) model with slit/cylindrical pore shape using nitrogen adsorption branch kernel.
